# Toward a humanized technology: a reinterpretation of Erich Fromm’ s humanistic peace psychology

**DOI:** 10.3389/fpsyg.2026.1646601

**Published:** 2026-02-16

**Authors:** Yun Wang, Peng Li, Wenyu Liu

**Affiliations:** 1 Institute of Marxism, Dalian University of Technology, Dalian, China; 2 School of Public Administration, Nanjing University, Nanjing, China; 3 School of Foreign Languages, Dalian University of Technology, Dalian, China

**Keywords:** artificial intelligence, autonomous weapons, digital media, Erich Fromm, humanized technology, peace psychology, surveillance

## Abstract

In contemporary society where technology has advanced significantly, the issues of war and peace are not only political or economic but also deeply tied to the structure of human psychology. Based on Erich Fromm’s humanistic psychology, this paper reexamines the adaptability and framework of peace psychology in the technological era. First, the paper explores the psychological roots of war, emphasizing that deep psychological mechanisms such as aggressiveness, idolization, and emotional numbness are key drivers of war. Second, the paper distinguishes between the concepts of “negative peace” and “positive peace,” proposing that positive peace should encompass positive psychological resources such as justice, love, and freedom, rather than merely the cessation of violent conflict. Furthermore, the paper analyzes the emotional coldness, numbness, and the aestheticization of war in the media brought about by technological alienation, revealing the erosion of the psychological foundation for peace by these technologies and proposing “humanized technology” as a solution. By emphasizing that technology should serve humanity’s empathy, creativity, and freedom, rather than alienation and control, this paper provides new perspectives for the theoretical innovation and practical development of contemporary peace psychology. The core contribution of this paper lies in the interdisciplinary integration of Fromm’s peace psychology with contemporary technological advancements, presenting strategies to address the impact of technology on individuals and society. Finally, the article provides important topics for future empirical research, including the psychological effects of emotional detachment and digital warfare, peace education in algorithmic environments, and the psychological and ethical studies of artificial intelligence and neural technologies.

## 1 Introduction

Technological acceleration has become a defining feature of contemporary social life and, increasingly, of contemporary war. Across the early 2020s, the diffusion of algorithmically curated platforms, militarized artificial intelligence (AI), and networked surveillance has altered how conflict is waged, represented, and psychologically processed. Studies of digital conflict communication and mediated suffering show that platform logics can compress attention, intensify emotional arousal, and transform distant violence into consumable spectacle, with ambiguous consequences for empathy and public judgement (Chouliaraki and Al-Ghazzi, 2022; Zhan, 2022). Related work on weaponized digital environments further suggests that algorithmic engagement incentives can amplify outrage and moral panic, complicating deliberation about war and peace (Pizzi et al., 2021).

Parallel literatures in security studies and AI ethics have highlighted the destabilizing potential of autonomy and decision-support systems in military contexts, including novel escalation pathways, shifts in responsibility, and risks to strategic stability (Dean, 2022; Renic, 2023; Montgomery and Nelson, 2025; Kühn and Williams, 2025). At the societal level, empirical research indicates that information segregation and affective polarization can be reinforced by platform architectures and algorithmic curation, even when causal effects vary by context (McLaughlin, 2025; Kuehn, 2023). These dynamics matter for peace psychology because they shape the emotional and cognitive conditions under which people interpret threats, attribute intentions, and recognize the humanity of outgroups (Guo, 2022).

In a different register, Erich Fromm’s humanistic psychoanalysis has experienced a visible scholarly revival in the 2020–2025 period (Cortina, 2015). Contemporary work reconsiders the ethical and psychological grounds of Fromm’s humanism, his attention to otherness, and the cultural conditions that shape character and consciousness (Layton, 2024; Romanetto, 2024; Cortina, 2024; Buechler, 2024; Over, 2021). This renewed scholarship is often motivated by contemporary crises—resurgent authoritarianism, widening inequality, ecological precarity—and by the search for conceptual resources capable of resisting dehumanization and cultural resignation (Frie, 2025).

Despite these advances, an important research gap remains. Analyses of emerging technologies and conflict typically proceed at the level of governance, strategic risk, or legal-ethical critique; they rarely examine the psychological and characterological mechanisms through which technicized environments cultivate indifference, destructiveness, and moral disengagement (Funk, 2025; Frie, 2025). Conversely, renewed Fromm scholarship clarifies the meaning of humanism and the dynamics of social character, but it has not yet been systematically connected to contemporary peace psychology or to the specific socio-technical architectures—algorithmic feeds, autonomous weapons, immersive media, and pervasive surveillance—that now mediate violence and political life. As a consequence, peace psychology lacks a coherent Frommian framework that translates humanistic ethics into concrete guidance for evaluating the peace-relevant effects of technology (Falcone et al., 2025).

To address this gap, the present article offers a conceptual reconstruction of Fromm’s humanistic peace psychology for the technological age. Building on Fromm’s distinction between biophilic and necrophilic orientations and his analysis of alienation, we articulate a peace-psychological account of “humanized technology”: socio-technical systems oriented toward sustaining relatedness, autonomy, and critical reason, rather than amplifying fear, dependency, and instrumental domination (Frie, 2024a). We then apply this framework to prominent technological domains that increasingly structure conflict and peace: mediated war spectacles, lethal autonomous weapons, digital surveillance, and brain–computer interfaces. Throughout, we integrate the negative/positive peace distinction to clarify how these technologies can obstruct not only the cessation of violence but also the cultivation of the psychological and cultural capacities that positive peace requires.

The remainder of the article is organized as follows. Section 1 outlines Fromm’s basic peace-psychological assumptions; Section 2 clarifies negative and positive peace; Section 3 analyses emerging technological threats to peace; and Section 4 develops a Frommian account of humanized technology and proposes implications for future research and practice in peace psychology.

This conceptual study makes three interrelated contributions relative to recent scholarship (2020–2025). First, it bridges renewed work in Fromm studies with contemporary peace psychology by operationalizing Fromm’s humanistic ethics and social-character analysis as analytic categories for assessing socio-technical environments. Recent Fromm scholarship has clarified the psychoanalytic grounds of Fromm’s humanism and its relevance for current crises (Cortina, 2024; Layton, 2024; Romanetto, 2024; Buechler, 2024), but it has seldom been translated into a peace-psychological framework oriented to technological mediation.

Second, it extends technology-and-war debates beyond questions of regulation and strategic stability by specifying the psychological mechanisms through which particular technologies can normalize violence: emotional numbing and voyeuristic spectatorship in mediated war consumption (Chouliaraki and Al-Ghazzi, 2022; Zhan, 2022), dehumanization and moral distancing in autonomous warfare (Asaro, 2012; Renic, 2023), and the erosion of agency and critical consciousness under digital surveillance (Murray, 2024).

Third, it articulates a constructive normative agenda. “Humanized technology” is defined not as a technological fix but as a set of humanistic design and governance commitments—supporting relatedness, enabling agency, and preserving moral responsibility—that can be evaluated empirically and refined through interdisciplinary work. This complements, but is conceptually distinct from, prevailing human-centered AI programs that foreground usability and safety (Shneiderman, 2020; Frie, 2024b; Putri, 2024) by insisting that peace is also a psychological project that requires cultivating biophilic orientations and resisting the commodification of human relations.

Through this interdisciplinary lens, this study seeks to demonstrate that Fromm’s humanism is not an obsolete legacy, but a radical remedy for the technological alienation and civilizational crises of the 21st century.

## 2 Basic theory of Fromm’s humanistic peace psychology

Fromm contends that a credible peace theory must be grounded in a developed theory of human nature—what he terms a humanistic science of man (Fromm, 1955, p. 12). In other words, understanding human nature—specifically, human needs, fears, and the potential for love and hate—is the prerequisite for understanding the possibility of peace (Fromm, 1961, p. 23).

### 2.1 War as a psychological issue

Erich Fromm (1900–1980), born into a Jewish family in Frankfurt, Germany, experienced the traumatic impact of two world wars and developed a profound understanding of the psychological scars war inflicts on both individuals and societies (Ahmad et al., 2023). The issue of war deeply influenced Fromm’s thought, especially his perplexity over why war continues to exist and why humans, despite yearning for peace, often support war (Clark and Friedman, 2015). In *Escape from Freedom*, Fromm explored the roots of fascism from a social-psychological perspective, revealing the role of psychological factors in war and violence (Fromm, 1941, p. 5). He argued that war is not merely a political or economic issue, but is driven by deep psychological mechanisms, particularly an irrational dependence on violence and power.

In the 1930s, Fromm conducted an empirical study in Germany to investigate the working class’s attitudes towards war. The responses to the question of “how to prevent a new world war” revealed fundamental differences in people’s views on war (Boehnke et al., 2005). Some conservatives believed war was an inherent part of human nature, a necessary expression of national power; fascists saw war as an inevitable aspect of social Darwinism; socialists argued that war was a product of capitalism’s inherent nature, and it could only be eliminated through social reform; while liberals advocated for the elimination of war through education, progress, and international cooperation (Fromm, 1941, p. 12). These differing perspectives show that the roots of war lie not only in material or political conditions but are deeply driven by psychological factors (Barbiero and Berto, 2021).

Fromm maintained that preventing war cannot rely solely on rational or material analysis, but requires psychological intervention to dissect the inner violent impulses of individuals and groups. He clearly asserted that violence and destructiveness are not innate human instincts but products of specific psychological and social conditions (Fromm, 1973, p. 4). This perspective provides a crucial psychological foundation for understanding the irrational dynamics of war.

### 2.2 Fromm’s theory of human nature and aggression

From a humanistic perspective, Fromm argued that war and violence are not innate human instincts but are distorted responses that arise when humans’ basic needs are inadequately met (Fromm, 1947, p.65). Fromm posited that humans not only have basic survival needs but also face five existential needs due to the “dichotomy of existence” (e.g., life and death, individualization and isolation): connection, transcendence, grounding, identity, and orientation. When these needs are satisfied in positive ways (e.g., through love, creativity, and rational self-actualization), they promote individual happiness and social peace, fostering positive forces such as life-enhancement, solidarity, justice, and creativity (Ryff and Singer, 2008). Conversely, when these needs are inadequately addressed or are met in regressive ways (e.g., necrophilia, sadism, destructiveness, greed, narcissism), they can give rise to harmful tendencies that become the psychological roots of conflict and violence (Fromm, 1973, p. 6).

In his 1973 work *The Anatomy of Human Destructiveness*, Fromm criticized the notion that humans have an inherent destructive impulse, calling this idea “obviously absurd.” He explicitly rejected Freud’s concept of the “death instinct” (Fromm, 1973, p. 4). Unlike Freud, Fromm distinguished between two types of aggression: one is biologically adaptive, such as “reactive aggression” arising from self-defense, which is a common phenomenon in nature; the other is “malignant aggression,” unique to humans and rooted in individual character and social pathology (Fromm, 1973, p. 9–10). He further noted that these forms of aggression are closely tied to the unique conditions of human existence.

One of Fromm’s key contributions is his profound analysis of the “inner tension” of human existence. Humans are part of nature, needing to satisfy basic biological needs, yet they can transcend nature through self-awareness, rationality, and imagination (Han, 2022). He frequently referenced the fundamental duality of human existence: “Humans can never escape two conflicting tendencies: one that leads them away from the maternal origin—moving from an animalistic state of existence toward a human state, toward freedom; the other leads them back to the maternal origin, back to nature, back to certainty and security” (Fromm, 1955, p. 34). This conflict between freedom (growth, individuality) and belonging (security, unity) constitutes the core tension in human psychological structure.

Due to the lack of the instinctive mechanisms and physiological adaptability that other species possess, humans are more vulnerable and anxious than other animals (Fromm, 1941, p. 36–39). Fromm argued that human infants are “the most helpless of animals,” requiring far more care than other species; adults, on the other hand, possess the ability to transcend animal instincts with imagination and rationality, allowing them to be aware of dangers that have not yet occurred, living in abstract concepts, history, and the future, rather than just in the present moment. Thus, humans tend to fear symbolic threats or uncertainties about the future, not just direct physical threats (Rios et al., 2018).

Furthermore, Fromm observed that humans tend to construct ideologies and self-identity, linking them to abstract values such as nationality, religion, ethnicity, and political ideologies. Attacks on these values are often experienced as attacks on one’s core self. This capacity makes human aggression more intense and destructive than the innate aggression of animals (Van Hiel et al., 2020).

### 2.3 Classification of destructive psychology: sadistic, necrophilic, and reactive aggression

In his exploration of human aggression and destructiveness, Fromm classified malignant (destructive) aggression into three main types: sadistic aggression, narcissistic (or collective narcissistic) aggression, and necrophilic aggression. These destructive personality types each possess distinct psychological structures, social causes, and philosophical foundations, and manifest in different forms in modern society. A deeper understanding of these types helps to uncover the psychological dynamics behind war and violence, providing valuable insights for the pursuit of peace (Drožđek et al., 2020).

#### 2.3.1 Sadistic aggression

Sadistic aggression arises from the desire for absolute control and domination over others—seeking pleasure through cruelty, torture, and humiliation to assert power (Papagathonikou and Marono, 2025). This form of aggression is a non-constructive destructive satisfaction, used to fill the individual’s void of meaning and strength. Fromm suggests that if an individual cannot create life or meaning, they may attempt to substitute this deficit by destroying life. Sadistic aggression is a “malignant aggression” rooted in distorted personality structures, unrelated to the life instinct, but driven by desires for control, domination, humiliation, and dehumanization of others. Unlike biological defensive aggression, sadistic aggression does not aim for survival but seeks absolute power, to control others’ will, and to degrade their personhood (Hardeck, 2025). Therefore, Fromm argues that understanding and resisting the spread of sadistic aggression is not only an individual psychological treatment task but also a necessary practice of cultural and social critique. This viewpoint embodies a key tenet of peace psychology: the irrational obsession with power and destruction is a root cause that prevents humanity from achieving true freedom and peace (Fromm, 1973, p. 6).

#### 2.3.2 Necrophilic aggression

Necrophilic aggression represents the most extreme and pathological form of destructive behavior (Murad et al., 2025). Fromm uses the concept of “necrophilia” to describe a pathological fascination with death and destruction (Pettigrew, 2022). In this context, necrophilia does not refer to a narrow sexual fetish but to a psychological preference for all things dead, mechanical, or lifeless. Fromm defines necrophilia as a “love for death,” where the individual’s orientation revolves around death, decay, and destruction. People with necrophilic tendencies are obsessed with violence, blood, and destruction, harboring a pathological fascination with death, desiring the “pleasure of killing,” and even being interested in corpses and torture (Itzkowitz, 2017). In this personality type, life is despised, death is worshiped, and destruction becomes an end in itself. Fromm argues that the philosophical basis for this psychological structure can be found in the concept of the “need for transcendence.” Humans have an inherent need to transcend their own finite existence, and the healthy way to achieve this is through creation, procreation, and love (Fromm, 1956, p.6). However, when an individual cannot transcend themselves in constructive ways, they may turn to destruction as a means of achieving a false sense of transcendence—gaining control over the ultimate mystery of life through death, either of themselves or others. Destruction becomes a substitute for creation: those unable to create life resort to destroying life, in a futile attempt to challenge life’s transience and their own insignificance (Fromm, 1973, p. 9).

#### 2.3.3 Reactive aggression

Reactive aggression is a defense mechanism shared by both humans and animals to respond to perceived threats. However, in humans, this aggression is amplified by consciousness, symbolism, and social identity (Boccadoro et al., 2021). Humans can be manipulated to feel that their “survival interests” or “core values” are threatened, even if these threats are not real. Fromm introduced the human-specific tendencies of “idol-making” and “idol-worship,” whereby people create and surrender to various idols, such as nations, races, religions, or even abstract concepts like freedom, democracy, socialism, or consumerism and technological progress (Fromm, 1981, p.19). When people’s worship of these idols reaches pathological extremes, any challenge or threat to the idol is perceived as an attack on their core identity (Fromm, 1973, p. 270). Fromm warned that this idolized sense of threat is a critical root of human hostility and destructiveness. When a group is taught to worship a particular nation or ideology and to view others as “enemies,” they are persuaded that war is not only necessary but even just. War is often rationalized as a “just act to defend freedom and democracy” or “to preserve national security.”

Thus, Fromm reminds us that to prevent war, it is essential to identify and critique these psychological mechanisms of idolization, paranoia, and manipulation. One mission of peace psychology is to reveal how these mechanisms foster hatred and violence, and to help people identify and resist false threats (Fromm, 1973, p. 270).

## 3 Negative peace and positive peace

### 3.1 Positive peace: harmonious coexistence between individuals, society, and nature

In peace studies, the distinction between “negative peace” and “positive peace” is a foundational concept (Galtung, 1969). Fromm emphasizes that peace is not merely the absence of violence and war but also includes positive states within social structures and interpersonal relationships. “Negative peace” refers to the absence of open violent conflict, with no wars or armed confrontations. In contrast, “positive peace” signifies a state of harmonious cooperation, justice, and comprehensive human development, characterized by harmony between individuals, between humanity and nature, as well as the full development of rationality and love (Bayerlein et al., 2024). This concept aligns closely with the Hebrew term “shalom” in the Old Testament, which refers to a state of wholeness, completeness, and well-being. Therefore, positive peace is not just the cessation of hostilities or the end of war but an environment that fulfills human needs and fosters mutual support and trust (Fromm, 1962, p. 20).

Fromm argues that only positive peace constitutes sustainable peace. Even if a society is in a state of non-conflict (i.e., “negative peace”), if it remains filled with injustice, oppression, or structural violence, it will eventually lead to the eruption of new conflicts (Hirschfeld, 2017).

### 3.2 Negative peace: the state of no war

Although Fromm advocates for positive peace, he also recognizes the urgency of his time, especially the destructive threat posed by nuclear weapons (Albulescu, 2023). In this context, Fromm argues that the primary step toward achieving positive peace is to ensure “negative peace”—the cessation of hostilities, as its alternative could be total destruction. As he stated, “Given the threat of total destruction, we no longer have time to wait for humanity and society to undergo profound transformations to achieve peace” (Fromm, 1962, p. 20).

During the Cold War, superpowers maintained a fragile peace based on the principle of “mutually assured destruction” (MAD), a negative peace grounded in threats (Wangh, 1986). Fromm expressed deep skepticism about this approach, arguing that living under the shadow of nuclear annihilation would have severe psychological effects on society. He noted, “Living under the constant threat of destruction for long periods will have a significant psychological impact on most people—it generates fear, hostility, indifference, rigidity, and a disregard for everything we hold dear” (Fromm, 1962, p. 23). While nuclear deterrence may temporarily prevent war, it erodes humanity’s fundamental understanding of peace. Fromm warned that defending freedom in this way might ultimately lead to the loss of freedom: “If we truly argue that our goal is to preserve freedom—to prevent humanity from becoming the vassal of an authoritarian state—we must realize that, regardless of whether nuclear deterrence succeeds or not, this freedom will be lost” (Fromm, 1962, p. 23).

Fromm emphasized that the greatest danger of unstable peace based on deterrence is the loss of human vitality and hope (Fromm, 1994, p.16). When society’s focus is excessively directed toward fear and threats, people neglect the importance of positive ideals and human development. He stated, “Freedom, individuality, and faith increasingly become empty slogans; creativity declines; our vision of a better life no longer transcends the ever-expanding consumer desires” (Fromm, 1968, p. 8). This spiritual numbness and degradation hinder the construction of a better future.

### 3.3 The psychological force of “shalom,” love, freedom, and hope

To meet these challenges, Fromm advocates cultivating a constructive social horizon. Even if a fully harmonious world remains distant, we should begin, here and now, to build solidarity, moral courage, and an affirmative outlook on peace (Hindle, 2025). In Fromm’s framework, *hope* constitutes the psychological and moral pillar of peace. In *The Revolution of Hope*, he calls for a “revolution of hope” in the face of catastrophic anxiety (Fromm, 1968, p. 9–10). He further argues that “history often shows that even an idea without the support of power can possess unexpected strength (Fromm, 1950, p.78). We need a new attitude—neither sentimental nor irrational—that nonetheless affirms the reality of possibility.” Hope is not fantasy, but confidence that human beings can change their conditions despite uncertainty. “Hope is the spiritual accompaniment of life and growth” (Fromm, 1968, p. 7).

Absent hope, individuals lapse into despair or numbness—especially when confronted with war, climate change, or entrenched inequality. Contemporary public discourse often privileges fear over hope; Fromm cautions that overemphasizing catastrophic narratives can paralyze collective agency. Peace psychology should therefore take hope as a central reference point, mobilizing collective action and enabling constructive peace-oriented shifts (Roque-Hernández, 2022).

Fromm also warns against an ascetic ideology increasingly visible in modern societies. In response to climate change and resource constraints, some propose “degrowth” or severe austerity as the sole path forward (Fromm, 1964, p. 16). While acknowledging the need for restraint and sustainability, Fromm argues for an inspiring, humane vision of the future—a warmer, more human-centered trajectory of growth. As he puts it, “We should adopt a perspective that emphasizes development in a different and more human way” (Fromm, 1976, p. 143). For Fromm, genuine positive peace is not merely the cessation of war, but the creation of social conditions in which human potential can flourish and life is no longer driven by fear, greed, or domination.

## 4 Emerging technological threats to peace

### 4.1 Fromm’s critique of technological society: indifference, alienation, and emotional numbing

Rapid advances in science and technology have transformed social life and, with it, the landscape of challenges and opportunities for peace (Kühn and Williams, 2025). Fromm consistently underscored a dual effect: technology can enhance human welfare and help reduce drivers of war—by alleviating scarcity or illuminating the psychology of aggression—yet unregulated progress may outpace moral development, generating new forms of alienation, indifference, and destructive power. In *The Revolution of Hope*—subtitled *Toward a Humanized Technology*—he argues that technology must be humanized, i.e., subordinated to human needs and values; otherwise it risks facilitating new wars rather than peace (Fromm, 1968).

Already in the mid-twentieth century, Fromm identified dangers that are even more salient today. He warned against celebrating technology as an end in itself while marginalizing the human, a stance akin to contemporary “techno-utopianism” or technophilia—the belief that complex social problems (or even “unmanageable” persons) can be solved by high-tech weapons or automated systems (Talmadge, 2019). Fromm locates this attitude in a *necrophilous orientation*—an attraction to the mechanical and inanimate that goes hand in hand with indifference to life (Fromm, 1973, p. 365). He also diagnosed an unhealthy split of affect and reason: “a mild but chronic schizophrenic state—a split between feeling and thinking”—whose consequence is not only the growth of hostility but a pervasive indifference to life; such indifference may be more dangerous than overt destructiveness because it underwrites the willingness to destroy others and oneself (Fromm, 1968, p. 53). As emotional numbing intensifies—when killing is equated with “pressing a button” or war is consumed through screens—individuals become more likely to perpetrate or tolerate atrocities. This numbing threatens peace more than open hatred by rendering violence dehumanized, routinized, and increasingly virtualized (Montgomery and Nelson, 2025).

### 4.2 The impact of new technologies on the perception of peace in the 21st century

#### 4.2.1 New media and the “entertainment” of war

In the digital era, Fromm’s warnings have become increasingly salient. The rise of networked media and real-time communication has immersed societies in a torrent of conflict content (Ruhl Ibarra et al., 2025). Rather than relying solely on state narratives, users are continuously exposed—via social platforms—to war reporting, videos, and commentary. This information deluge is typically fragmented and decontextualized, and it is often packaged for attention capture in sensory, gamified formats. Such dynamics generate “information fog” and overload, obscuring ground truth and weakening critical appraisal (Kraitzman et al., 2025).

Across television and social media (e.g., X/Twitter), coverage frequently foregrounds tactical moves, territorial gains and losses, and looping explosions, while effacing the human costs of war. As coverage comes to resemble sports broadcasting—with spectators tracking “who is up” on the battlefield, accompanied by nationalistic cheers—images of civilian suffering are quickly subsumed by strategic analysis or state-centered frames (Chouliaraki and Al-Ghazzi, 2022). The result is diminished empathic capacity: audiences mourn less, identify less with victims, and are more likely to experience satisfaction when the “home side” prevails. This power-game narrative encourages emotional disengagement from war’s realities and appears not only in tabloid formats but at times within mainstream outlets.

Fromm would regard these narrative forms as amplifying societal “psychic numbness” and affective decline. When war is packaged as reality entertainment or a strategy game, the public’s moral imagination—the capacity to place oneself in the other’s position—is blunted, undermining the constituency for peace (Fromm, 1968, p. 53).

The core implication in Fromm’s framework is that such numbness cannot be accepted as normal; it must be actively countered. Peace psychology in the technological age therefore faces two linked questions: How do virtualized violence and remote warfare (e.g., drones, cyber operations) reshape cognition and affect? And how can empathic concern and a sense of shared humanity be sustained in technologically mediated environments? For Fromm, the more automated and “surgical” violence appears, the more dangerous it becomes, because the presentation severs the spontaneous empathic responses that ordinarily restrain destructive impulses (Fromm, 1968, p. 53).

Consider the operator launching a missile from a distant control room, or the civilian viewing night-vision footage of bombardment: neither is confronted with the visceral experience of death and suffering on the ground. This distance and abstraction produce an “emotional vacuum” (Zhan, 2022). The task for peace psychology is to help fill that vacuum—by re-humanizing distant others, educating about the real consequences of war, and cultivating media practices that promote global empathy rather than antagonism.

#### 4.2.2 Lethal autonomous weapons (LAWs): dehumanization

Lethal autonomous weapons (LAWs) are AI-driven systems capable of independently identifying and striking targets, executing violence without human emotional engagement (Asaro, 2012). Fromm argues that the normalization of violence progressively dulls individuals’ responses to it (Fromm, 1973, p. 63). This insight illuminates the threat posed by LAWs: when the execution of lethal force is no longer modulated by human emotion and moral judgment but by impersonal algorithms, violence becomes dehumanized (Renic, 2023). Fundamental respect for life and empathic concern are stripped away, and violent action is rendered “rational” and “normal” (Wagner, 2014).

As Fromm writes, once violence is detached from affective and ethical regulation, it risks becoming a social norm (Fromm, 1941, p. 20). Such dehumanized violence fosters numbness in both individuals and society toward war and conflict. By shifting life-and-death decisions to machine algorithms, LAWs remove the moral shock that would otherwise accompany the use of force, reframing violence as a “reasonable” course of action (Dean, 2022). In turn, societal and individual acceptance of violence increases, thereby weakening the psychological motivations that sustain peace.

#### 4.2.3 Surveillance technologies: the erosion of freedom

Surveillance technologies—especially facial recognition, data tracking, and large-scale monitoring—significantly erode individual freedom and privacy (Stoycheff, 2016). Fromm argues in *Escape from Freedom* that freedom is central to human existence and that its loss stems not only from external control but also from an inner atrophy of vital energies (Fromm, 1941, p. 35). When virtually every action and decision is subject to external observation, personal autonomy is constrained (Penney, 2017). Fromm further warns that chronic control and the anxiety it induces can produce emotional numbing, diminishing sensitivity to injustice and violence and fostering a state akin to learned helplessness (Fromm, 1941, p. 22).

This numbing weakens motivation to pursue peace. Surveillance not only engenders a sense of powerlessness in the face of social wrongs, it also heightens perceived external threat, encouraging conformity and dependence on prevailing power structures and thereby undermining the willingness to speak and act in contexts of conflict (Murray, 2024). As Fromm contends in *The Revolution of Hope*, ongoing control and insecurity tend to dull affect and sap initiative, leading individuals to relinquish efforts to change the status quo (Fromm, 1968, p. 64). The diffusion of surveillance thus reduces collective sensitivity to violence and injustice, impedes mobilization, and attenuates the psychological foundations that sustain peace.

#### 4.2.4 Brain–computer interfaces (BCIs): crises of autonomy and identity diffusion

Brain–computer interfaces (BCIs) couple neural activity directly to external devices to augment cognition or control machines (Sun and Ye, 2023). Such tight coupling raises distinctive risks for personal autonomy: when external systems can shape or co-determine thought and action, the individual’s capacity for self-directed agency may be compromised, intensifying psychological alienation (Fromm, 1973, p. 64).

Fromm treats freedom and autonomy as core conditions of mental health; interference by external forces in the domain of thought and conduct undermines this inner freedom and erodes the bases of responsible choice (Fromm, 1973, p. 136). In the BCI context, blurred boundaries between “self” and “machine” can destabilize identity formation, producing self-doubt and estrangement (Gilbert et al., 2019). This self-alienation, which Fromm analyzes as a hallmark of modernity’s flight from freedom, weakens deep interpersonal attachment and diminishes the motivational foundations of empathy and prosocial commitment essential to peace.

Accordingly, without robust psychological–ethical safeguards—e.g., protections for agency, informed consent, reversibility, and meaningful human control—BCIs risk normalizing forms of heteronomy that erode the subjective capacities on which peace-oriented cognition and behavior depend (Steinert et al., 2019).

## 5 Toward humanized technology: Fromm’s insights for contemporary peace psychology

### 5.1 The contemporary value of Fromm’s peace psychology

Fromm’s core claim is that durable peace is not merely the absence of war but the realization of inner freedom, reason, love, and empathy. Peace, in this view, is propelled by transformations in character structure: when individuals overcome fear, isolation, and destructive impulses, social harmony becomes possible (Wilson, 2024). This framework retains strong relevance amid today’s psychosocial crises shaped by technologically mediated alienation.

Contemporary violence extends beyond interstate warfare to terrorism, racial conflict, and discontent rooted in widening inequalities (Cheliotis, 2011). Fromm argues that violence is not a fixed human instinct but emerges when social environments and existential needs fall out of balance (Fromm, 1944). Such conflicts are amplified when unmet needs thwart communication and empathy, fracturing social bonds and fueling antagonism.

While modern technologies have expanded productivity and convenience, they also intensify emotional distancing and interpersonal estrangement. For Fromm, technology ought to serve basic human needs—freedom, love, creativity—rather than become an instrument of domination or alienation (Fromm, 1968, p. 61–62). In digital ecosystems, platform logics and information silos can attenuate empathic responsiveness and normalize “virtual” forms of hostility, reinforcing the very psychic conditions that undermine peace (Fromm, 1968, p. 53).

Accordingly, peace must be approached as a psychological as well as political project: cultivating inner freedom, affective attunement, and mature rationality is indispensable to counteracting destructive tendencies and to institutionalizing humane modes of conflict resolution. On this basis, Fromm’s humanistic peace psychology remains a timely resource for addressing the emotional numbing and social fragmentation characteristic of the technological age (McLaughlin, 2015).

### 5.2 Humanizing technology: advancing contemporary peace psychology

The accelerating diffusion of artificial intelligence, big data analytics, unmanned systems, and automated weapons is reshaping social interaction and psychological functioning (Shneiderman, 2020). Technology is not intrinsically malign; it extends human needs and capacities. Yet Fromm warned that when technological development is uncoupled from human interior needs, it becomes a force of alienation (Fromm, 1971). In the digital era, such alienating effects are increasingly visible: pervasive social media, fragmented information flows, and the ubiquity of algorithmic systems strain affective life, moral judgment, and practical reason, allowing instrumental rationality to dominate and rendering relationships more objectified—conditions conducive to emotional blunting and social estrangement (Jobin et al., 2019).

Fromm’s notion of “humanized technology” offers a normative compass for peace psychology: socio-technical systems should be designed to serve core human needs—freedom, creativity, and love—rather than to intensify control or alienation (Kühn, 2019). Embedding these needs in technology governance can counteract psychosocial fragmentation and strengthen social cohesion (Floridi et al., 2018). The stakes are acute in domains such as AI-enabled and autonomous weapons, where dehumanized decision chains can normalize violence and mechanize warfare, dulling empathic and moral restraints (Fromm, 1973, p. 53). Accordingly, peace psychology should help specify ethical guardrails and psychosocial design criteria so that technological applications promote relational health and nonviolent problem solving (Hagendorff, 2020).

Humanizing technology also requires sustained attention to how systems shape affect, cognition, and moral perception. Education and policy should cultivate ethical responsibility and empathic competencies for life in digital environments, positioning technology as a tool for peacebuilding rather than a catalyst of division (Murad et al., 2024). This agenda entails not only technical regulation but also interventions at the level of psychological development and social institutions, thereby advancing a contemporary peace psychology capable of addressing technology-driven forms of alienation (Pizzi et al., 2021).

### 5.3 Practical pathways for advancing contemporary peace psychology

To advance peace psychology, interventions should be designed at three interlocking levels—individual psychology, social structures, and strategic policy orientation. At the individual level, Fromm foregrounds love and reason as core capacities for peace. In response to affective blunting and psychological alienation intensified by technology and globalization, education, culture, and social policy should prioritize affective education, critical judgment, and responsibility (Kester and Cremin, 2017). Beyond policy levers, targeted psychological interventions and cultural practices can strengthen resistance to violence and war and cultivate prosocial orientations (Cremin, 2015).

At the societal level, Fromm’s call for “humanized technology” underscores that technical development should not be guided solely by efficiency and control but must also foster relational connectedness, social responsibility, and mental health (Halperin et al., 2011). Stakeholders across sectors—industry, government, and academia—should co-develop technology ethics standards and assessment frameworks to ensure that socio-technical systems do not exacerbate social division or psychological estrangement but instead support inclusion and wellbeing (Shneiderman, 2020).

At the policy level, the strategic orientation should shift from defeating adversaries to making enmity unnecessary through coexistence and mutual gain. Fromm cautions that conceiving the end of war as victory or humiliation of the other is both insufficient and dangerous: if the objective is the opponent’s destruction, any ensuing “peace” will be short-lived (Bahador, 2012). Post-conflict arrangements must be designed for coexistence among parties that must continue to share the world. Peace strategies should avoid cornering the adversary, which tends to radicalize positions, empower hardliners, and convert moderates into hawks. The viable pathway is to recognize shared interests—survival, security, dignity, and economic wellbeing—and to replace zero-sum logics with win-win solutions. Rather than demonizing the “enemy,” policymakers should invest in dialogue and perspective-taking to elicit and affirm the other’s humanity (Krieg and Xu, 2023). Where interlocutors within the opposing camp still hold humane values or have not succumbed to fanaticism, they can become partners for engagement. This orientation aligns with contemporary conflict-resolution practices that emphasize empathy, narrative exchange, and trust-building (Kelman, 2005).

## 6 Conclusion

This article reinterprets Erich Fromm’s peace psychology in the context of 21st-century technological developments and proposes new theoretical directions and frameworks. It reconstructs Fromm’s peace psychology, emphasizing the humanistic, destructive psychology, and the theory of technological alienation. By integrating Fromm’s analyses of emotional numbness, alienation, and necrophilic tendencies with contemporary technologies (such as artificial intelligence, unmanned warfare, social media algorithms, and surveillance technologies), the article reveals how new technologies are leading to the depersonalization of warfare, the entertainment of war reporting, and the dissolution of fear, cruelty, and reverence for violence, ultimately fostering destructive psychological tendencies. This reconstruction not only offers a fresh perspective on understanding the psychological crises induced by modern technologies but also highlights how humanistic peace psychology can address the alienating effects of technology on individuals and society.

As technological advancements—particularly in artificial intelligence, big data, and automation—continue to evolve, peace psychology must actively respond to their profound impact on human psychology and social relationships. The article advocates for the “humanization of technology,” emphasizing that technology should serve humanity’s emotional, creative, and free capacities, rather than becoming a tool for oppression and alienation. As Fromm proposed in *The Revolution of Hope* (1968), technology should be directed toward promoting human flourishing, not enslaving humanity. By pursuing this path, peace psychology can offer effective interventions to counteract the negative psychological effects of technology, while fostering a more harmonious social environment.

This study also provides a comprehensive agenda for future empirical research. Future studies could explore the psychological effects of emotional detachment and the normalization of violence in automated warfare and drone use. Another area for research is the development of peace education in algorithmically influenced environments, investigating how to nurture empathy and rational judgment among global citizens in the context of fragmented and virtualized information. Additionally, research on the psychological ethics of artificial intelligence and neurotechnologies should be prioritized, with a focus on ensuring ethical practices and protecting individual autonomy.

In conclusion, this article not only restates the core ideas of Fromm’s peace psychology but also offers new interpretations in light of contemporary technological contexts, advancing theoretical innovation and practical development in the field of peace psychology. Through deepening these theories and fostering interdisciplinary integration, peace psychology retains broad relevance and significance in the technological age.
